# Digital and traditional recruitment strategies and participation patterns in the multicenter Healthy Brain Ageing study

**DOI:** 10.3389/fdgth.2026.1862779

**Published:** 2026-08-05

**Authors:** Daniel F. Pilco-Janeta, Philipp Mahlknecht, Myriam De la Cruz-Puebla, Corinne Horlings, Alicia Garrido, Taina M. Marques, Ruxandra Soare, Christoph Theyer, Simon Leiter, Soumyabrata Ghosh, Kavita Rege, Iris Egner, Jón Pol Gales, Sebastian Schade, Andrés Granolles, Fernanda Farfan, Venkata P. Satagopam, Claudia Trenkwalder, Brit Mollenhauer, Rejko Krüger, Werner Poewe, Eduardo Tolosa, María-José Martí

**Affiliations:** 1 Parkinson's Disease and Movement Disorders Unit, Neurology Service, Hospital Clínic de Barcelona, Barcelona, Spain; 2 Department of Neurology, Medical University of Innsbruck, Innsbruck, Austria; 3 Institut de Neurociències, Universitat de Barcelona, Barcelona, Spain; 4 Centro de Investigación Biomédica en Red Sobre Enfermedades Neurodegenerativas (CIBERNED) CB06/05/0018-ISCIII, Barcelona, Spain; 5 Transversal Translational Medicine, Luxembourg Institute of Health, Strassen, Luxembourg; 6 Luxembourg Centre for Systems Biomedicine, University of Luxembourg, Esch-sur-Alzette, Luxembourg; 7 Paracelsus-Elena-Klinik Kassel, Kassel, Germany; 8 Digital Marketing Agency, Altoclick, Barcelona, Spain; 9 Department of Neurosurgery, University Medical Center Goettingen, Goettingen, Germany; 10 Department of Neurology, University Medical Center Goettingen, Goettingen, Germany; 11 Centre Hospitalier de Luxembourg, Luxembourg; 12 Lab of Parkinson's & Other Movement Disorders, Institut d'Investigacions Biomèdiques August Pi I Sunyer (IDIBAPS), Barcelona, Spain

**Keywords:** dissemination strategies, health communication, online health survey, Parkinson's disease, participant recruitment strategies

## Abstract

**Background:**

Early detection of Parkinson's disease (PD) benefits from innovative approaches such as large-scale online surveys on different risk factors in the general population. Effective dissemination is essential, yet little is known about the relative reach and recruitment patterns of different strategies.

**Objective:**

To describe dissemination strategies used in the multicenter Healthy Brain Ageing (HeBA) study and characterize center-specific recruitment-source, web-access, demographic, and survey-completion patterns across three European centers.

**Methods:**

Data from three centers (Barcelona, Spain; Luxembourg, Luxembourg; and Innsbruck, Austria) aiming at remotely identifying PD risk factors and prodromal symptoms were analyzed. Dissemination strategies were categorized as digital or traditional. Descriptive statistics summarized dissemination strategies, geographic web-access patterns, linguistic adaptation, recruitment sources, and survey completion.

**Results:**

At the campaign level, digital dissemination predominated in Barcelona (55.7%) and Luxembourg (53.3%), whereas Innsbruck used a slightly higher proportion of traditional campaigns (52.2%). Participant-reported recruitment sources showed center-specific patterns: digital advertising was the most frequent reported source in Barcelona (62.5%), flyers or invitation letters in Luxembourg (39.3%), and radio or television in Innsbruck (31.4%). Sex-based recruitment differences were significant in Luxembourg (*p* = 0.006) and Innsbruck (*p* = 0.009), and age-based differences were significant in all centers (Barcelona *p* < 0.001, Luxembourg *p* < 0.001, Innsbruck *p* = 0.001), with 82.0% of participants younger than 65 years. The proportion of participants in the high questionnaire-completion category (≥90% of items answered) was 77.1% in Barcelona, 94.5% in Luxembourg, and 92.8% in Innsbruck.

**Conclusions:**

Recruitment-related patterns differed across centers and reflected locally implemented combinations of digital and traditional dissemination channels. These findings suggest that recruitment strategies for large-scale online health studies may benefit from considering local media ecosystems, language contexts, and digital participation patterns.

## 1 Introduction

Parkinson's disease (PD) affects millions worldwide (1, 2) and significantly impacts motor function, cognitive abilities, and overall quality of life (3, 4). Early detection and intervention are important for improving patient outcomes and developing effective therapies. The European multicenter Healthy Brain Ageing (HeBA) study aims to identify individuals at risk for PD and other neurodegenerative conditions through a comprehensive online questionnaire (5–7). This questionnaire assesses various risk and prodromal factors, including non-motor symptoms, medical history, and lifestyle factors, which are associated with the development of PD (8–10). Given the scale and objectives of the HeBA study, dissemination was implemented across different geographic, linguistic, and media contexts. This multicenter study, conducted across research centers in Kassel, Germany; Innsbruck, Austria; Barcelona, Spain; and Luxembourg, Luxembourg, comprised two phases: Phase 1, including an online questionnaire (1a) and olfactory testing (1b), and Phase 2, involving in-person evaluations. The present analysis includes Barcelona, Innsbruck, and Luxembourg centers, which employed both digital and traditional dissemination strategies with comparable campaign-level records, participant-reported recruitment-source data, and web analytics. Kassel used a primarily postal-based recruitment strategy and was therefore not included in this descriptive analysis of mixed dissemination approaches.

PD is strongly age-associated, and population-based screening efforts therefore commonly focus on later adulthood (11). The HeBA rationale is grounded in evidence that neurodegeneration and prodromal features can precede clinically manifest motor PD by many years (5–7). The prodromal phase refers to the period in which early symptoms or signs of PD-related neurodegeneration are present but a clinical diagnosis based on fully developed motor parkinsonism cannot yet be made (8–10). Identifying individuals during this phase is important for characterizing early disease trajectories and for assembling cohorts suitable for future prevention or disease-modifying trials.

This study focused on Phase 1 of HeBA, describing dissemination strategies in these three centers. The objective was to describe dissemination approaches across Barcelona, Innsbruck, and Luxembourg and characterize observed recruitment-source, demographic, web-access, and survey-completion patterns. Using a retrospective observational design, this analysis describes center-specific patterns across different European recruitment contexts.

## 2 Methods

This retrospective, multicenter observational study analyzed HeBA data from Barcelona, Innsbruck, and Luxembourg collected between April 2022 and June 2024, corresponding to the completed recruitment period included in this report. Dissemination activities were locally adapted across centers. Recruitment-related patterns were assessed using campaign records, landing-page access metrics, participant-reported recruitment sources, survey records, and survey-completion data.

### 2.1 Data acquisition and management

Data were obtained from three sources: (1) the HeBA online survey, (2) records of communication campaigns, and (3) web analytics. These sources represented different analytical levels: campaign records captured dissemination activities, web analytics described anonymous visits to the study landing page, and survey records described participant-level questionnaire responses, including self-reported recruitment sources and survey completion. The online questionnaire was implemented using a web-based survey platform. Each exported record contained platform-generated identifiers, including a HeBA ID and HeBA token. Participants were aged 50 years or older and had no previous diagnosis of PD or another neurological disease. The age eligibility criterion was emphasized in all recruitment materials. The core questionnaire domains were common across centers, with local adaptations in language and recruitment-source response format. Individual-survey data included survey-record access dates and self-reported sources of study information. These access dates were used to describe temporal patterns of survey records. Because Barcelona used a single-response item and Luxembourg and Innsbruck allowed multiple responses, all responses were harmonized into common categories (Supplementary Table S1). Consequently, recruitment-source findings were interpreted as center-specific response distributions rather than directly comparable estimates of individual-level exposure.

Communication campaign data were compiled from internal records maintained by each center and included information on campaign characteristics such as campaign name, timing, media channel, and campaign type. Geographic patterns of initial access to the HeBA landing page were assessed using anonymized visit data collected through Matomo. All analyses were performed using the HeBA dataset version released on October 9, 2025.

### 2.2 Definition of digital and traditional media strategies and how participants learn about the survey

Recruitment methods were categorized as digital or traditional media (Supplementary Table S2). Digital methods included online or electronically delivered strategies, and traditional methods included print, broadcast, postal, or in-person approaches. These categories were used as broad descriptive groupings.

For participant-level Digital/Traditional analyses, records with multiple recruitment-source responses were classified as Digital or Traditional when all selected sources belonged to the same category. Records combining Digital and Traditional sources were classified as Mixed and excluded from the binary Digital versus Traditional analysis.

### 2.3 Ethical considerations

This study was conducted in accordance with the principles of the Declaration of Helsinki (12) and received approval from the ethics committees of all participating centers. All participants provided digital informed consent. The study was registered under the WHO-conform registry number DRKS00025979.

### 2.4 Statistical analysis

Statistical analyses were performed using Python (version 3.12.7) and R (version 4.5.0; R Foundation for Statistical Computing, Vienna, Austria). In Python, analyses and visualizations were conducted using pandas, matplotlib, seaborn, SciPy, and statsmodels. In R, spatial analysis and geovisualization were performed using dplyr, sf, rnaturalearth, and ggplot2.

Descriptive statistics summarized dissemination strategies, campaigns distribution, communication channels, and target languages. Pearson chi-square tests were applied to assess associations between dissemination method (digital or traditional) and participant sex, and age group with Phi and Cramér's V as effect size measures. Expected cell counts were inspected for the main chi-square analyses; no expected cell counts were below 5. Because of the large sample size, *p*-values were interpreted together with Phi or Cramér's V. Survey completion was calculated as the percentage of questionnaire items answered within each survey record and categorized as <25%, 25%–89%, or ≥90%. Survey completion categories (<25%, 25%–89%, ≥90%) were compared between centers using a chi-square test of independence with Cramér's V as effect size, followed by exploratory pairwise chi-square tests with Bonferroni-adjusted significance thresholds. The same chi-square framework was applied to evaluate associations between survey completion categories and sex and age group, both overall and stratified by center. 95% confidence intervals for proportions were estimated using Wilson score intervals. Statistical significance was set at *p* < 0.05. A Sankey diagram was used to illustrate the flow of communication methods, and maps were used to visualize geographic web-access patterns.

## 3 Results

### 3.1 Dissemination strategies

#### 3.1.1 Dissemination strategies across study sites

**Figure 1:**
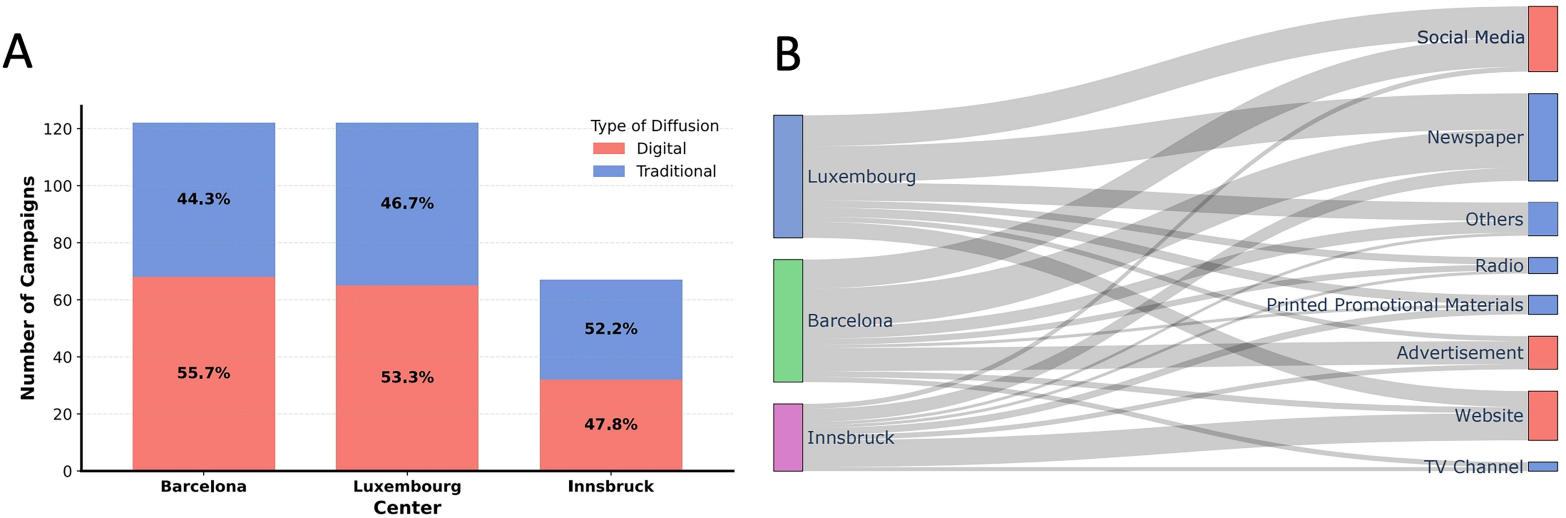
Overview of dissemination strategies across study centers. **(A)** Distribution of Digital and Traditional dissemination campaigns in Barcelona, Luxembourg, and Innsbruck. **(B)** Sankey diagram showing the specific communication channels used by each center (Barcelona, Luxembourg, Innsbruck). Digital methods (red) include social media, websites, and digital advertisements; Traditional methods (blue) include newspapers, radio, and TV.

At the campaign level, digital campaigns represented the largest proportion of dissemination activities in Barcelona (55.7%) and Luxembourg (53.3%), whereas Innsbruck used a slightly higher proportion of traditional campaigns (52.2%) (Figure 1A, Table 1).

**Table 1:** Distribution of dissemination media and campaign types across HeBA centers.

Type of Dissemination	Media	Barcelona *n* (%)	Luxembourg *n* (%)	Innsbruck *n* (%)
Traditional	Newspaper	38 (31.2%)	36 (29.5%)	13 (19.4%)
	Radio	6 (4.9%)	7 (5.7%)	3 (4.5%)
	TV Channel	5 (4.1%)	0 (0.0%)	4 (6.0%)
	Printed Promotional Materials	3 (2.5%)	9 (7.4%)	7 (10.4%)
	Others (Traditional)	11 (9.0%)	13 (10.7%)	3 (4.5%)
	Subtotal Traditional	54 (44.3%)	57 (46.7%)	35 (52.2%)
Digital	Social Media	29 (23.8%)	31 (25.4%)	5 (7.5%)
	Digital Advertisement	23 (18.9%)	5 (4.1%)	5 (7.5%)
	Website	6 (4.9%)	16 (13.1%)	27 (40.3%)
	E-mail	1 (0.8%)	5 (4.1%)	0 (0.0%)
	Subtotal Digital	68 (55.7%)	65 (53.3%)	32 (47.8%)
Total		122 (100.0%)	122 (100.0%)	67 (100.0%)

Distribution of dissemination media by center, type, and frequency of use, showing each HeBA center's distribution of Digital and traditional media channels. Counts represent dissemination actions or media-use records compiled from center campaign logs. Percentages indicate the relative frequency of each medium within its respective center.

In Barcelona and Luxembourg, social media advertising led (23.8% and 25.4%, respectively). In contrast, Innsbruck's digital strategy was dominated by different websites (40.3%). Across all three centers, newspaper outreach was a key component of the traditional campaigns, accounting for 31.2% of efforts in Barcelona and 29.5% in Luxembourg.

A Sankey diagram (Figure 1B) visualizes the flow of dissemination channels from each center to specific media types, highlighting each center's prioritization of strategies to engage the target population.

#### 3.1.2 Language distribution in HeBA dissemination strategies and participant response

Dissemination campaigns were tailored to the linguistic context of each center to ensure accessibility (Table 2). In Barcelona, dissemination primarily used bilingual Spanish and Catalan materials (44.8%). Luxembourg displayed a multilingual approach, with German (36.4%), French (28.1%), English (14.1%), and Portuguese (9.1%) as predominant languages. In contrast, Innsbruck exclusively used German.

**Table 2:** Language distribution in HeBA campaigns across centers.

Center	Language	*n* (%)
Barcelona	ES,CAT	52 (44.9%)
	ES	46 (39.7%)
	CAT	18 (15.5%)
Luxembourg	DE	44 (36.4%)
	FR	34 (28.1%)
	EN	17 (14.1%)
	PT	11 (9.1%)
	LU	7 (5.8%)
	FR,DE	5 (4.1%)
	FR,DE,PT,EN,LU	3 (2.5%)
Innsbruck	DE	67 (100.0%)

Distribution of languages used in HeBA dissemination campaigns by center, showing each language in Barcelona, Luxembourg, and Innsbruck. The languages are represented as follows: ES (Spanish), CAT (Catalan), DE (German), FR (French), EN (English), LU (Luxembourgish), and PT (Portuguese). Percentages indicate the relative frequency of language usage per center.

To describe language patterns among respondents, we analyzed the language of survey completion by recruitment channel (Supplementary Table S3). In Barcelona, participants assigned to digital recruitment mainly responded in Spanish (89.4%), whereas those assigned to traditional recruitment mostly used Catalan (70.2%). In Luxembourg, German was the most frequent response language for both digital (45.5%) and traditional (47.0%) recruitment. All Innsbruck participants responded in German, consistent with monolingual campaigns.

### 3.2 Recruitment reach and participation outcomes

#### 3.2.1 Geographic distribution of web access

**Figure 2:**
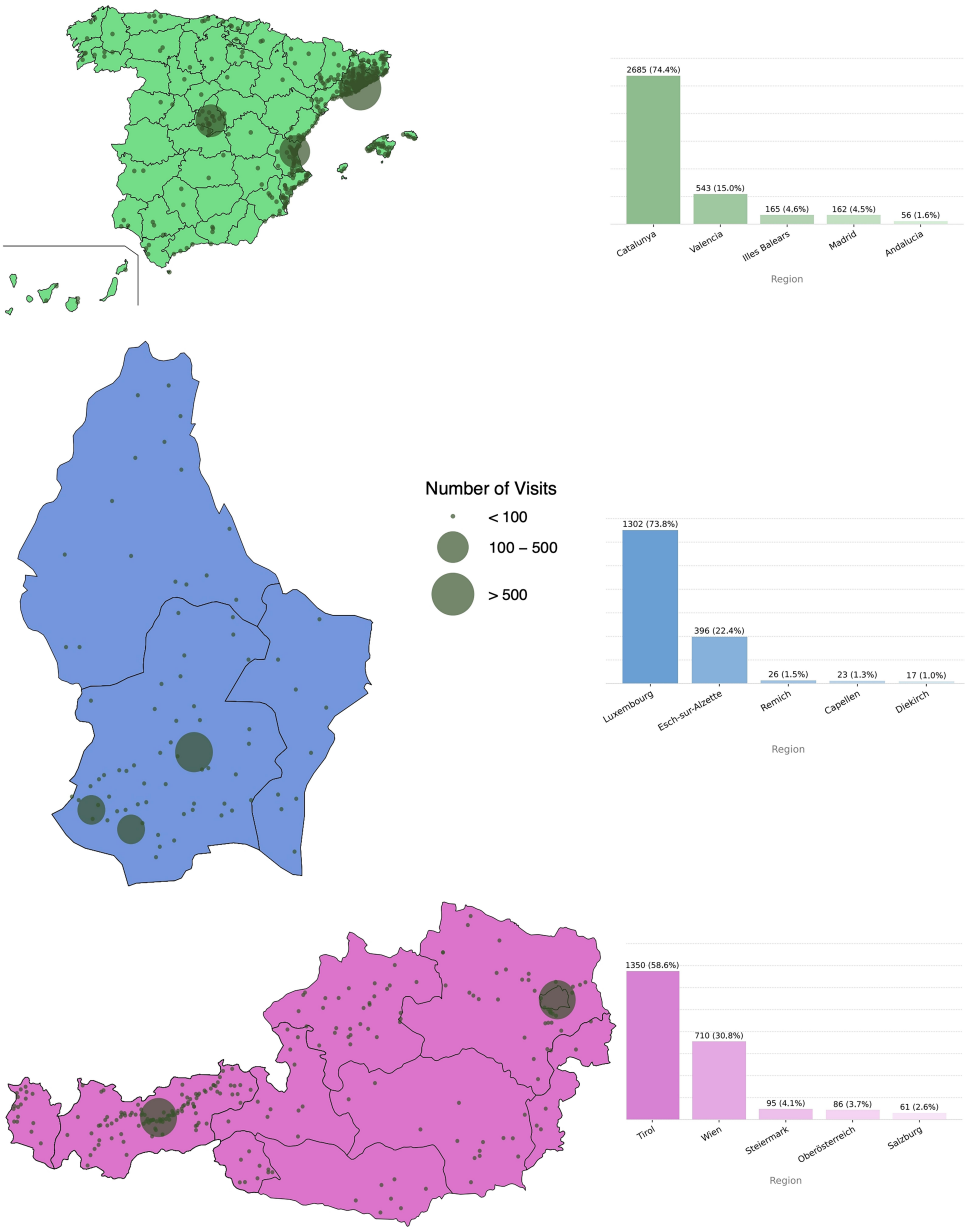
National distribution of web visits to the HeBA survey landing page across centers. National distribution of initial, anonymous web visits to the HeBA survey landing page. Each center is represented by a distinct color: green for Barcelona, blue for Luxembourg, and pink for Innsbruck. Dot sizes reflect the frequency of visits from each location: small dots indicate fewer than 100 visits, medium dots represent 100–500 visits, and large dots indicate over 500 visits. The bar charts emphasize the five regions with the most frequent initial interest in each country.

Matomo analytics were used to map anonymous visits to the study landing page across the three centers (Figure 2). In Spain, the highest number of accesses came from the autonomous communities of Catalonia (74.4%), followed by Valencia (15.0%) and the Balearic Islands (4.6%). In Luxembourg, the Capital Region accounted for the majority of web visits (73.8%), with the East of the Alzette region contributing 22.4%. In Austria, Tyrol (58.6%) and Vienna (30.8%) accounted for the majority of web visits.

#### 3.2.2 Participation metrics

Participant-level analytical denominators are summarized in Supplementary Figure S1. The analytic dataset included 10,062 survey records from Barcelona, 8,998 from Luxembourg, and 2,611 from Innsbruck. Completion analyses included 10,050, 8,959, and 2,608 records, respectively.

Temporal patterns of survey-record access dates showed peaks in Luxembourg in December 2023 (4,815 survey records). Barcelona and Innsbruck showed notable peaks in April 2023 (1,775 and 733 survey records, respectively) and in April 2022 (728 survey records in Innsbruck) (Supplementary Figure S2).

Table 3 presents participant-reported sources of information about the HeBA survey. Because Barcelona used a single-response format whereas Luxembourg and Innsbruck allowed multiple responses, results should be interpreted as center-specific response distributions. In Barcelona, digital advertising was the most frequent reported source (62.5%), followed by newspapers (22.4%) and in-person communication (8.8%). In Luxembourg, flyers or invitation letters were the most frequent reported source (39.3%), followed by email notifications (19.2%), newspapers (15.6%), and digital advertising (14.4%). In Innsbruck, radio or television was the leading reported source (31.4%), followed by newspapers (28.8%) and digital advertising (16.1%).

**Table 3:** Participant-reported sources of information about the HeBA survey across Barcelona, Luxembourg, and innsbruck.

How did you find out about this study?	Barcelona	Luxembourg	Innsbruck
Newspaper	1,357 (22.4%)	1,049 (15.6%)	505 (28.8%)
Radio/Television	190 (3.1%)	210 (3.1%)	551 (31.4%)
In-person communication	533 (8.8%)	494 (7.4%)	227 (12.9%)
Flyer or invitation letter	4 (0.1%)	2,636 (39.3%)	75 (4.3%)
Digital advertising	3,788 (62.5%)	967 (14.4%)	282 (16.1%)
Email	47 (0.8%)	1,288 (19.2%)	38 (2.2%)
Other	142 (2.3%)	65 (1.0%)	75 (4.3%)
Total	6,061 (100.0%)	6,709 (100.0%)	1,753 (100.0%)

Barcelona used a single-response format, whereas Luxembourg and Innsbruck allowed multiple responses. Percentages represent the proportion of total recruitment-source responses within each center. In Luxembourg and Innsbruck, multiple selections were split into separate response counts.

#### 3.2.3 Completeness patterns of online survey respondents

Survey completion was categorized as high (≥90%), moderate (25%–89%), and low (<25%) (Table 4).

**Table 4:** Completion level and participant characteristics across centers.

Variable	Subgroup	Total n	Completed < 25%	Completed 25% - 89%	Completed ≥ 90%	Chi-square (df)	p-value	Cramér's V
Global analysis	—							
Center		21,617				1,315.8 (4)	<.001	0.174
	Barcelona	10,050	452 (4.5%)	1,849 (18.4%)	7,749 (77.1%)			
	Innsbruck	2,608	31 (1.2%)	157 (6.0%)	2,420 (92.8%)			
	Luxembourg	8,959	91 (1.0%)	401 (4.5%)	8,467 (94.5%)			
Sex (Overall)		21,617				162.7 (2)	<.001	0.087
	Female	12,804	347 (2.7%)	1,714 (13.4%)	10,743 (83.9%)			
	Male	8,813	227 (2.6%)	693 (7.9%)	7,893 (89.6%)			
Age Group (Overall)		21,617				134.7 (6)	<.001	0.056
	50–59	8,517	238 (2.8%)	850 (10.0%)	7,429 (87.2%)			
	60–69	8,847	188 (2.1%)	951 (10.7%)	7,708 (87.1%)			
	70–79	3,860	115 (3.0%)	529 (13.7%)	3,216 (83.3%)			
	≥80	393	33 (8.4%)	77 (19.6%)	283 (72.0%)			
Barcelona	—							
Sex		10,050				67.2 (2)	<.001	0.082
	Female	6,748	275 (4.1%)	1,386 (20.5%)	5,087 (75.4%)			
	Male	3,302	177 (5.4%)	463 (14.0%)	2,662 (80.6%)			
Age Group		10,050				105.7 (6)	<.001	0.073
	50–59	3,734	169 (4.5%)	608 (16.3%)	2,957 (79.2%)			
	60–69	4,204	154 (3.7%)	762 (18.1%)	3,288 (78.2%)			
	70–79	1,891	99 (5.2%)	413 (21.8%)	1,379 (72.9%)			
	≥80	221	30 (13.6%)	66 (29.9%)	125 (56.6%)			
Luxembourg	—							
Sex		8,959				9.2 (2)	0.010	0.032
	Female	4,522	52 (1.1%)	229 (5.1%)	4,241 (93.8%)			
	Male	4,437	39 (0.9%)	172 (3.9%)	4,226 (95.2%)			
Age Group		8,959				24.2 (6)	<.001	0.037
	50–59	3,612	55 (1.5%)	162 (4.5%)	3,395 (94.0%)			
	60–69	3,669	24 (0.7%)	146 (4.0%)	3,499 (95.4%)			
	70–79	1,582	10 (0.6%)	87 (5.5%)	1,485 (93.9%)			
	≥80	96	2 (2.1%)	6 (6.2%)	88 (91.7%)			
Innsbruck	—							
Sex		2,608				1.7 (2)	0.427	0.026
	Female	1,534	20 (1.3%)	99 (6.5%)	1,415 (92.2%)			
	Male	1,074	11 (1.0%)	58 (5.4%)	1,005 (93.6%)			
Age Group		2,608				8.1 (6)	0.231	0.039
	50–59	1,171	14 (1.2%)	80 (6.8%)	1,077 (92.0%)			
	60–69	974	10 (1.0%)	43 (4.4%)	921 (94.6%)			
	70–79	387	6 (1.6%)	29 (7.5%)	352 (91.0%)			
	≥80	76	1 (1.3%)	5 (6.6%)	70 (92.1%)			

Completion categories defined as < 25%, 25%–89%, and ≥ 90% of items answered. Associations were tested using Pearson's chi-square with Cramér's V for effect size. Bonferroni correction was applied for global (3 tests, *α*=0.0167) and stratified (6 tests, *α*=0.0083) analyses. From an initial sample of *n* = 21,671, a total of *n* = 32 participants were excluded due to missing age data. Analyses by sex further exclude *n* = 22 participants who did not identify as male or female, resulting in a final analysis sample of *n* = 21,617.

Overall, most records included in the completion analysis reached the high-completion category (≥90%), with differences observed between centers (*χ*² = 1,315.8, df = 4, *p* < 0.001, Cramér's V = 0.174). Luxembourg showed the highest proportion of high-completion records (94.5%), followed by Innsbruck (92.8%) and Barcelona (77.1%). Low-completion surveys (<25%) were rare (≤4.5%).

Completion was significantly associated with sex (*χ*² = 162.7, df = 2, *p* < 0.001, V = 0.087) and age (*χ*² = 134.7, df = 6, *p* < 0.001, V = 0.056). Overall, a higher proportion of records from men reached high completion than records from women (89.6% vs. 83.9%). High completion was lowest among participants aged ≥80 years (72.0%).

By center, sex differences were most pronounced in Barcelona (men 80.6% vs. women 75.4%) (*χ*² = 67.2, *p* < 0.001, V = 0.082); weaker in Luxembourg (*χ*² = 9.2, *p* = 0.010, V = 0.032) and non-significant in Innsbruck (*p* = 0.427). Across centers, the lowest high-completion proportions were observed among participants aged ≥80 years.

#### 3.2.4 Demographic profile of participants by dissemination methods

**Figure 3:**
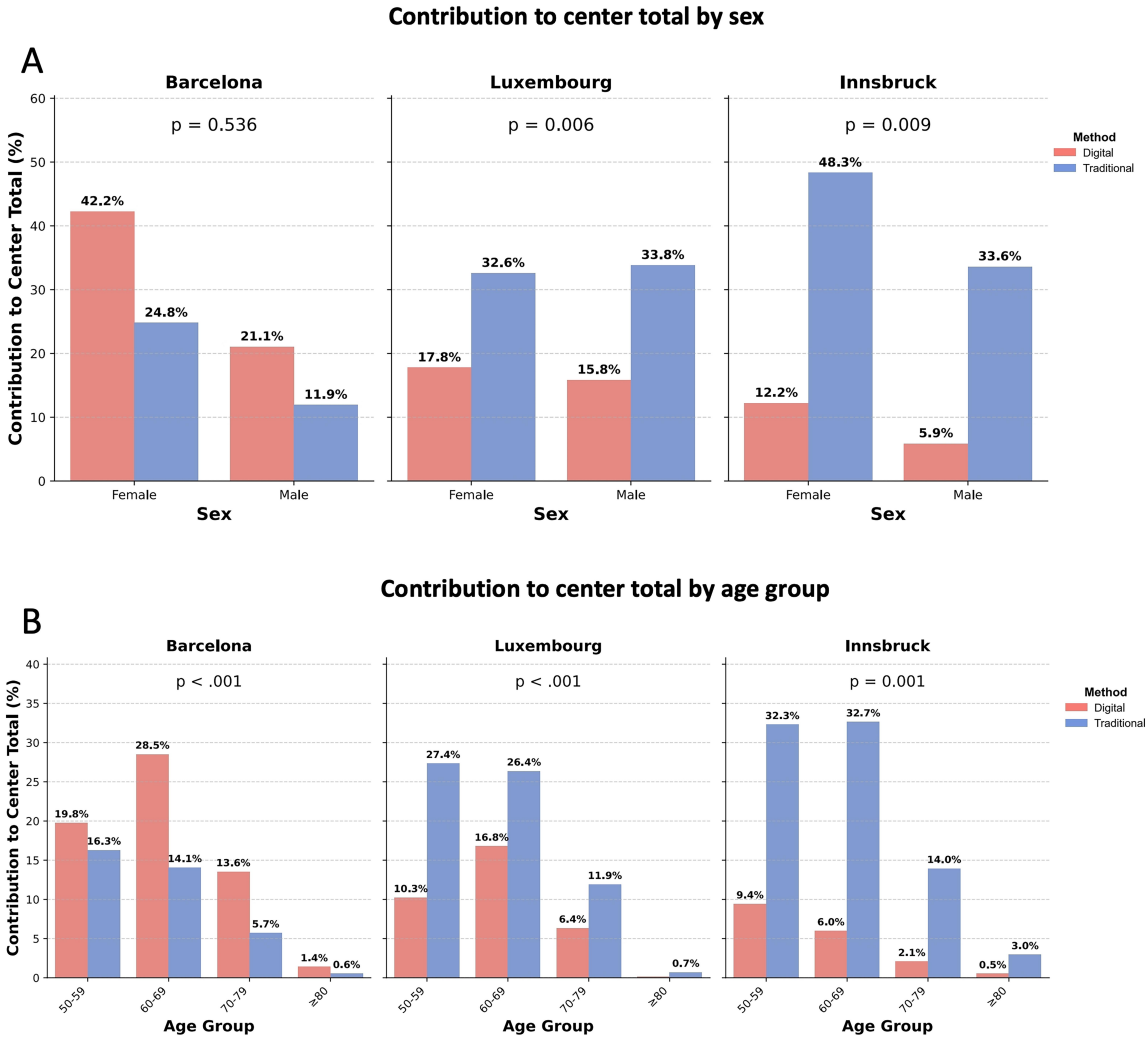
Demographic composition by recruitment method and center. Grouped bar charts showing the absolute contribution of each combination of recruitment method (digital in salmon, traditional in blue) and demographic subgroup to the total number of participants in each center. Panel **(A)** displays contributions by sex; panel **(B)** by age group. Values above bars indicate percentages of the center total, and *p*-values above each panel correspond to Pearson's chi-square tests assessing the association between recruitment method and the demographic variable (sex or age group) within each center.

Demographic analyses by recruitment method were restricted to records with available recruitment-source information that could be assigned to Digital or Traditional categories and with valid sex and age data (Supplementary Figure S1). Digital and traditional recruitment methods contributed differently to participants' demographics across centers (Figure 3 and Supplementary Table S4). Figure 3 presents the contribution of each method-demographic combination to the center analytical total, whereas Supplementary Table S4 presents the Digital/Traditional distribution within each demographic subgroup to support interpretation of the association tests.

Recruitment method was not associated with sex in Barcelona [*χ*²(1) = 0.38, *p* = 0.536, *φ* = 0.008]. Associations were observed in Luxembourg [*χ*²(1) = 7.62, *p* = 0.006, *φ* = 0.036] and Innsbruck [*χ*²(1) = 6.73, *p* = 0.009, *φ* = 0.067], although effect sizes were small.

Recruitment method was associated with age group in Barcelona [*χ*²(3) = 110.64, *p* < 0.001, Cramér's V = 0.135], Luxembourg [*χ*²(3) = 77.05, *p* < 0.001, V = 0.114], and Innsbruck [*χ*²(3) = 15.47, *p* = 0.001, V = 0.102]. Supplementary Table S4 shows that the relative distribution of Digital and Traditional recruitment differed across age groups within each center.

## 4 Discussion

### 4.1 Dissemination and web-access patterns

This study described dissemination strategies employed across three HeBA centers (Barcelona, Innsbruck, and Luxembourg) for recruiting participants to an online survey for Parkinson's disease (PD) risk. The findings describe center-specific patterns in dissemination strategies, participant-reported recruitment sources, web access, participant characteristics, and survey completion. Recruitment relied on a combination of traditional and digital communication channels. Newspaper, radio and television have been commonly used in similar studies (13, 14) and continue to be part of dissemination efforts. At the same time, the inclusion of digital tools such as social media and website-based materials reflects recent trends observed in other studies (15–19), which note their ability to support wider and faster access to information.

Web access was concentrated mainly within the regions where recruitment activities were conducted, namely Catalonia in Spain, the capital region in Luxembourg, and Tyrol in Austria. These patterns likely reflect the regional scope of dissemination efforts. Matomo web visits were interpreted as indicators of initial web access rather than participant-level recruitment outcomes. Several factors may contribute to the observed center-specific patterns. Traditional channels, including postal invitations and in-person or locally embedded networks, may remain particularly relevant for reaching older adults or geographically defined populations (20). In contrast, digital channels may facilitate rapid web access, although participation in online research involves additional steps including website access, digital consent, and questionnaire completion (21). These requirements may influence which individuals ultimately become represented in the analytical sample.

### 4.2 Language and local implementation

Language selection in the dissemination campaigns reflected the sociolinguistic context of each center. This approach aimed to maximize participation in the HeBA survey. Barcelona used bilingual Spanish and Catalan, Luxembourg employed a multilingual strategy, including not only its three official languages (French, German, and Luxembourgish) but also Portuguese and English to specifically engage with its large immigrant communities, reducing potential participation barriers. In contrast, Innsbruck's campaigns were conducted exclusively in German, aligning with the linguistic homogeneity of the Tyrol region. In Barcelona, recruitment-source patterns also differed by response language. Participants reporting digital recruitment mainly completed the survey in Spanish, whereas those reporting traditional recruitment mainly completed it in Catalan. One possible explanation is that traditional dissemination in Barcelona was mainly implemented through local media in Catalonia, including Catalan-language radio and television, while digital dissemination was implemented more broadly across Spain and primarily used Spanish, although Catalan-language materials were also available. Thus, response language may reflect both the language context of the campaign and the participant's selected survey language. This pattern is consistent with the role of language preference in health information-seeking (22), although the study was not designed to test this mechanism directly.

In Luxembourg, the mix of languages was consistent across both channels, primarily reflecting the everyday use of German and French, while also reaching English and Portuguese-speaking communities.

### 4.3 Recruitment-source and temporal patterns

Survey participation varied temporally across centers. Peaks in Innsbruck and Barcelona (April 2022 and April 2023, respectively) coincided with World Parkinson's Day, when a combination of digital and traditional dissemination campaigns was conducted. This temporal alignment suggests that event-focused outreach may increase engagement (23, 24). In Luxembourg, the largest increase in web access occurred after the distribution of invitation letters, suggesting that direct mail may have been an important local recruitment pathway in this setting (20). However, because exposure denominators and conversion rates were unavailable, this pattern should not be interpreted as evidence of comparative channel effectiveness.

Analysis of how participants learned about the survey highlights center-specific recruitment patterns. Digital advertising dominated in Barcelona, flyers or invitation letters in Luxembourg, and radio or television in Innsbruck. In Luxembourg, this center-specific pattern is consistent with previous work showing that non-digital or traditional recruitment approaches can remain useful for reaching some older adult populations (25, 26). These findings underscore the need to tailor dissemination to local information ecosystems and cultural norms (27, 28).

### 4.4 Survey completion and demographic patterns

Completion rates were high among records included in the completion analysis. However, completion reflects the percentage of questionnaire items answered and should not be interpreted as a comprehensive measure of data quality or participant engagement (29). Differences between centers may reflect a combination of contextual factors such as dissemination mode, language context, and digital familiarity across populations (30, 31). High-completion records were common in all three centers. The higher proportions in Luxembourg and Innsbruck may be related to how participants were recruited. In Barcelona, most participants learned about the study through digital advertising, whereas invitation letters were more common in Luxembourg and radio or television were more common in Innsbruck. Participants reached through letters or broadcast media may have been more motivated to complete the survey, although motivation was not measured in this study. Completion patterns varied modestly by age and sex, with older participants showing lower completion, highlighting the importance of accessible and user-friendly designs for ageing populations (31). Although the HeBA study targeted adults aged 50 years and older, the achieved sample was concentrated mainly among middle-aged and younger-old adults. Because participation required digital consent and online questionnaire completion, lower completion among older participants may reflect lower digital access or confidence, as well as physical or cognitive limitations that can make online participation more difficult (32–34). These factors were not directly assessed in the present analysis.

The analysis of demographic characteristics by dissemination method showed center-specific patterns in the composition of the analytical sample. Female participation was generally higher across all centers, consistent with previous voluntary health studies (14, 35), but the contribution of each recruitment method varied. In Barcelona, digital recruitment contributed more than traditional recruitment to both female and male participant totals. Although digital advertising in Barcelona allowed audience targeting by age and sex, this was not sufficient to fully balance the final sample. In Luxembourg and Innsbruck, traditional methods accounted for the majority. These patterns suggest that recruitment channels may interact with local media use and health-information-seeking behaviors.

Age-related patterns also differed. In Barcelona, digital recruitment contributed most among participants aged 60–69 years, while traditional methods in Luxembourg contributed most among participants aged 50–69 years. These findings are consistent with previous observations that non-digital outreach can remain useful in certain population groups (25, 26, 36). Innsbruck showed a uniform pattern, with traditional channels contributing most across all age groups. Together, these findings suggest the value of multimodal, locally adapted recruitment strategies, particularly when aiming to include older adults or groups less likely to complete online questionnaires (35, 36).

Overall, these findings indicate that no single dissemination strategy is universally optimal. Instead, observed recruitment patterns appear to depend on how well dissemination approaches align with the local linguistic, cultural, and media context of each center. Combining digital and traditional channels may help support locally adapted recruitment, but comparative channel effectiveness could not be directly estimated in this retrospective analysis. Future studies would benefit from prospectively tracking exposure pathways and participant flow to further refine the evaluation of dissemination strategies in multicenter settings.

### 4.5 Strengths and limitations

This analysis has several strengths. It included a large number of survey records from three European centers with different linguistic, cultural, and media contexts. It combined campaign records, web analytics, participant-reported recruitment sources, demographic data, and survey-completion information, allowing recruitment to be described at several levels. The use of common Digital and Traditional categories supported a consistent analytical framework across centers, while center-specific analyses avoided assuming that recruitment patterns were identical across settings. The study also reports effect sizes and analytical denominators, which improves interpretation and transparency.

Several limitations should be noted. First, this retrospective descriptive analysis did not include comparable exposure denominators or conversion metrics across recruitment channels. Therefore, it cannot estimate the comparative effectiveness of individual channels. Exposure denominators indicate how many people had the opportunity to encounter a recruitment activity, while conversion metrics describe how many progressed from exposure to website access, consent, survey initiation, and completion. These measures were not consistently available across centers and channels.

Second, participants may have been exposed to multiple dissemination channels. Self-reported source of information should therefore be interpreted as a reported recruitment pathway, not definitive attribution to a single channel.

Third, Matomo web analytics were used to describe anonymous geographic web access, not recruitment outcomes. These data depended on cookie acceptance, repeated visits by the same person may have been possible, and geolocation may be approximate. Therefore, these data were interpreted as a complementary indicator of early online interest, separate from participant-level analyses.

Fourth, the recruitment-source item differed across centers: Barcelona used a single-response format, whereas Luxembourg and Innsbruck allowed multiple responses. Although responses were harmonized, recruitment-source percentages should be interpreted as center-specific response distributions.

Fifth, the Digital/Traditional distinction was used as a broad descriptive framework. Each group included heterogeneous channels, and these categories should not be interpreted as homogeneous recruitment modes.

Finally, all participation required digital consent and online questionnaire completion, which may have underrepresented people with limited digital access or confidence. Missing recruitment-source data may also have affected the demographic analyses by recruitment method because participants without this information could not be included in that specific analysis, although they remained included in other analyses for which the required data were available.

## 5 Conclusions

This retrospective multicenter analysis showed that dissemination strategies, participant-reported recruitment sources, web-access patterns, demographic composition, and completion differed across HeBA centers. The findings suggest the value of multimodal, locally adapted recruitment strategies for large-scale online studies of brain ageing and prodromal Parkinson's disease. Future digital health studies should prospectively track recruitment pathways and offer hybrid or assisted participation options to improve inclusion of older or less digitally confident groups.

## Data Availability

The datasets presented in this article are not readily available because the data analyzed in this study are available upon reasonable request from the HeBA data access committee via the first or corresponding author. Requests to access the datasets should be directed to Dr. María-José Martí (mjmarti@clinic.cat).

## Group member of HeBA consortium

Ilenia Bonini, Emna Bouhajja, Monika Buchner, Myriam De la Cruz-Puebla, Atbin Djamshidian, Iris Egner, Alessandra Fanciulli, Fernanda Farfan, Jon Gales, Alicia Garrido, Soumyabrata Ghosh, Valentin Groues, Somaye Hajian-Tilaki, Horst Hakelberg, Beatrice Heim, Sahra Henze, Corinne Horlings, Sonja Jonsdottir, Florian Krismer, Rejko Krüger, Elisabeth Lang, Simon Leiter, Philipp Mahlknecht, Kathrin Marini, Maria J Martí, Brit Mollenhauer, Ulf Nehrbass, Alastair Noyce, Claire Pauly, Noelia Peña Arauzo, Daniel F Pilco-Janeta, Werner Poewe, Regina Rautenkranz, Kavita Rege, Venkata Satagopam, Susanne Scaglione, Sebastian Schade, Sabine Schmitz, Reinhard Schneider, Susana Schnell, Anette Schrag, Katharina Schwarzová, Klaus Seppi, Cristina Simonet, Ruxandra Soare, David Stahl, Maritta Starke, Heike Stockner, Christoph Theyer, Eduardo Tolosa, Claudia Trenkwalder, Ludmilla Vasilev, Carlos Vega, Liliana Vilas Boas, Tamara Wicke, Laura Zamarian.

## Supplementary material

Supplementary Figure S1 Analytical denominator flow by center. Survey records correspond to individuals who accessed the study website, provided consent, and generated a survey record in the analytical dataset. Completion analysis included records with the information required to calculate survey completion. Recruitment-source analysis included records with non-missing participant-reported source-of-information data. Digital/Traditional analyses were restricted to records that could be assigned to one of the two broad recruitment-source categories.

Supplementary Figure S2 Temporal distribution of survey-record by center. Monthly distribution of survey records by access date and completion category across the three centers. The number above each bar indicates the number of survey records for that month.
