# Supplementary material for: Digital and traditional recruitment strategies and participation patterns in the multicenter Healthy Brain Ageing study
Source: Front Digit Health. 2026 Aug 5;8:1862779. doi: 10.3389/fdgth.2026.1862779 (PMC13486224; doi:10.3389/fdgth.2026.1862779)
Supplement: Supplementary file 1 [file Table1.docx]

**Supplementary Tables:**

**Supplementary Table S1: Harmonization of Recruitment Source Options**

| **Harmonized category** | **Original options in the HeBA survey** |
| --- | --- |
| Newspaper | Newspaper article, Newspaper advertisement, Local magazine/periodical |
| Radio/Television | Radio report, Television report/interview |
| In-person communication | In-person communication, Doctor, Patient/Seniors' Association |
| Flyer or invitation letter | Flyer, Invitation letter |
| Digital advertising | Online paid advertisements, social media (e.g., Facebook, Twitter) |
| Email | Email* |
| Other | Other (including unclassified free-text responses) |

Detailed breakdown of the original recruitment source options provided in the HeBA survey and their harmonization into standardized categories for analysis.

*Email was not provided as a distinct pre-defined option in Barcelona; however, responses mentioning "email" in the free-text "Other" field were manually recoded into this category.

**Supplementary Table S2: Definition of Digital and Traditional Dissemination Strategies**

| Category | Dissemination Methods Included |
| --- | --- |
| Digital Media | Paid advertising on social media (Meta, Google Ads), organic social media campaigns, outreach via Digital news portals, and targeted emails to specific cohorts and to patients subscribed to hospital newsletters. |
| Traditional Media | Outreach through radio and television reports/interviews; Outreach through newspapers and specialized magazines; Mass mailings of flyers or generic invitation letters; Distribution of personalized invitation letters; In-person events and word-of-mouth promotion; Outdoor advertising |

Definition of the digital and traditional media categories used for the classification of dissemination strategies. The table includes all strategies used in the HeBA project; however, implementation varied by center, and not all methods were applied uniformly across sites.

**Supplementary Table S3: Language of Participant Response by Recruitment Strategy**

| **Center** | **Language** | **Digital** | **Traditional** |
| --- | --- | --- | --- |
| Barcelona | Catalan | 401 (10.6%) | 1554 (70.2%) |
|  | Spanish | 3367 (89.4%) | 659 (29.8%) |
| Luxembourg | English | 369 (18.2%) | 787 (20.4%) |
|  | French | 712 (35.2%) | 1206 (31.3%) |
|  | German | 922 (45.5%) | 1808 (47.0%) |
|  | Portuguese | 22 (1.1%) | 48 (1.2%) |
| Innsbruck | German | 266 (100.0%) | 1203 (100.0%) |

Data show the language used by participants for enrollment, broken down by diffusion type and center. Values are presented as n (%). Percentages represent the proportion within each recruitment type per center.

**Supplementary Table S4**

Recruitment method within demographic subgroups by center

| **Characteristic** | **Subgroup** | **Barcelona** | | **Luxembourg** | | **Innsbruck** | |
| --- | --- | --- | --- | --- | --- | --- | --- |
|  |  | **Digital, n (%) [95% CI]** | **Traditional, n (%) [95% CI]** | **Digital, n (%) [95% CI]** | **Traditional, n (%) [95% CI]** | **Digital, n (%) [95% CI]** | **Traditional, n (%) [95% CI]** |
| **Sex** | Female | 2559 (63.0% [61.5–64.5]) | 1501 (37.0% [35.5–38.5]) | 1050 (35.3% [33.6–37.0]) | 1926 (64.7% [63.0–66.4]) | 181 (20.2% [17.7–22.9]) | 716 (79.8% [77.1–82.3]) |
|  | Male | 1275 (63.8% [61.7–65.9]) | 722 (36.2% [34.1–38.3]) | 936 (31.9% [30.2–33.6]) | 1999 (68.1% [66.4–69.8]) | 87 (14.9% [12.2–18.0]) | 498 (85.1% [82.0–87.8]) |
|  | *Association statistics* | *χ²(1) = 0.38, p = 0.536, φ = 0.008* | | *χ²(1) = 7.62, p = 0.006, φ = 0.036* | | *χ²(1) = 6.73, p = 0.009, φ = 0.067* | |
|  |  |  |  |  |  |  |  |
| **Age group** | 50-59 | 1198 (54.8% [52.7–56.9]) | 988 (45.2% [43.1–47.3]) | 606 (27.3% [25.4–29.1]) | 1617 (72.7% [70.9–74.6]) | 140 (22.6% [19.5–26.1]) | 479 (77.4% [73.9–80.5]) |
|  | 60-69 | 1729 (67.0% [65.1–68.8]) | 853 (33.0% [31.2–34.9]) | 993 (38.9% [37.0–40.8]) | 1560 (61.1% [59.2–63.0]) | 89 (15.5% [12.8–18.7]) | 484 (84.5% [81.3–87.2]) |
|  | 70-79 | 821 (70.2% [67.5–72.8]) | 348 (29.8% [27.2–32.5]) | 376 (34.8% [32.0–37.7]) | 705 (65.2% [62.3–68.0]) | 31 (13.0% [9.3–17.9]) | 207 (87.0% [82.1–90.7]) |
|  | ≥80 | 86 (71.7% [63.0–79.0]) | 34 (28.3% [21.0–37.0]) | 11 (20.4% [11.8–32.9]) | 43 (79.6% [67.1–88.2]) | 8 (15.4% [8.0–27.5]) | 44 (84.6% [72.5–92.0]) |
|  | *Association statistics* | *χ²(3) = 110.64, p < .001, V = 0.135* | | *χ²(3) = 77.05, p < .001, V = 0.114* | | *χ²(3) = 15.47, p = 0.001, V = 0.102* | |

*Note.* Percentages are calculated within each center-specific sex or age subgroup, using participants with an available recruitment source assignable to Digital or Traditional and valid data for the corresponding demographic variable. Digital and Traditional percentages therefore sum to 100% within each subgroup, apart from rounding. Confidence intervals are Wilson 95% confidence intervals. Pearson chi-square tests were performed separately by center; φ is reported for sex and Cramér’s V for age group.
